# Another Famous Man Saw Spirits

**Published:** 1892-07

**Authors:** 


### ANOTHER FAMOUS MAN SAW SPIRITS.

Walter Besant, a famous English author, "Secretary of the Palestine Exploration Fund;" educated at King's College, London, and Christ's College, Cambridge, England, gaining high mathematical honors; author of many successful novels and plays, and other valuable and able works, writes to the *Pall Mall Budget* the following:

My personal experience of spooks is not much, but it is, perhaps, more than falls to the lot of most. The first "figure" I ever saw was about six o'clock on an evening in September. I had been writing up to the last moment of daylight; it became too dark for me to see any longer and I knocked off. As I turned from the window I became aware that a female figure was in the room; it made no sign, but it moved about noiselessly. As I looked it disappeared. I was then living as a bachelor in chambers, and my outerdoor was closed so that no one could be in the room except myself.

Another experience, and a far more singular one, was this. I was traveling in Northumberland. The day I had spent in driving over a wild and lonely moor to a village set in the midst of it—a village built round the quadrangle of what had been a monastery. There was the old gate left; part of the buildings; part of the wall; the quiet village inclosed by the old wall; the convent chapel, now the parish church; there were only two or three hundred people living here; outside ran and babbled the trout stream with its high bank covered with bushes and brambles and wild flowers. All round stretched the moor. At the inn, where I took some tea or something, they talked to me about the past; the place was filled with echoes of the past; whispers and voices were heard at night; things had been seen in the bedrooms.

A wonderful place; no where else in England is there such a wonderful place. I drove back and spent the evening alone in my inn, reading certain books of the Queen Anne time, and at 11 o'clock went off to bed. My room was a very old room, and the inn itself was at least three hundred years old.

All this is introduction in order to show you why the thing that I saw took the shape that it did. For in the middle of the night I woke up suddenly and sat up startled. I found the room perfectly light; the door, which I had locked, flew open, and there walked in three ladies, dressed in the Queen Anne costume, with the pretty old stiff cardboard ornament of the head and everything. Never before had I understood how beautiful was the Queen Anne dress. The ladies sitting down on chairs round the fire (which was now burning merrily) began to talk, but I know not what they said. Suddenly—it shames me to confess the thing—I was seized with a horrid terror. I leaped from the bed, pulled back the curtains and pulled up the blind. It was about three in the morning, and twilight. Then I turned to my visitors; they slowly faded away. The light slowly went out of the room; the fire slowly burned low; the figures slowly became faint; they slowly vanished. Who were they? Well. You see that I have seen things.—*Exchange.*

